# Editorial: Music and the Functions of the Brain: Arousal, Emotions, and Pleasure

**DOI:** 10.3389/fpsyg.2018.00113

**Published:** 2018-02-09

**Authors:** Mark Reybrouck, Tuomas Eerola, Piotr Podlipniak

**Affiliations:** ^1^Musicology Research Unit, KU Leuven, Leuven, Belgium; ^2^Department of Music, Durham University, Durham, United Kingdom; ^3^Institute of Musicology, Adam Mickiewicz University in Poznan, Poznan, Poland

**Keywords:** music, functions of the brain, arousal, emotions, pleasure-pain principle

Music impinges upon the body and the brain and has inductive power, relying on both innate dispositions and acquired mechanisms for coping with the sounds. This process is partly autonomous and partly deliberate, but multiple interrelations between several levels of processing can be shown. There is, further, a tradition in neuroscience that divides the organization of the brain into lower and higher functions. The latter have received a lot of attention in music and brain studies during the last decades. Recent developments in neuroimaging techniques, however, have broadened the field by encompassing the study of both cortical and subcortical processing of the sounds. Much is still to be investigated but some major observations seem already to emerge. The domain of music and emotions is a typical example with a major focus on the pleasure that can be derived from listening to music. Pleasure, however, is not the only emotion that music can induce and the mechanisms behind its elicitation are far from understood. There are also mechanisms related to arousal and activation that are both less differentiated and at the same time more complex than the assumed mechanisms triggering basic emotions. It is tempting, therefore, to bring together contributions from neuroscience studies with a view to cover the possible range of answers to the question what pleasurable or mood-modifying effects music can have on human beings in real-time listening situations.

These questions were the starting point for a special research topic about music and the functions of the brain that was launched simultaneously in Frontiers in Psychology and Neuroscience. Scientists working on music from separate disciplines such as neuroscience, musicology, comparative musicology, ethology, biology, psychology, evolutionary psychology, and psychoacoustics were invited to submit original empirical research, fresh hypothesis and theory articles, and perspective and opinion pieces reflecting on this topic. Articles of interest could include research themes such as arousal, emotion and affect, musical emotions as core emotions, biological foundation of aesthetic experiences, music-related pleasure and reward centers in the brain, physiological reactions to music, automatically triggered affective reactions to sound and music, emotion and cognition, evolutionary sources of musical sensitivity, affective neuroscience, neuro-affective foundations of musical appreciation, cognition and affect, emotional and motor induction in music, brain stem reflexes to sound and music, activity changes in core emotion networks triggered by music, and potential clinical and medical-therapeutic applications and implications of this knowledge. The response to the call for papers yielded a wealth of proposals with 11 accepted papers by 43 contributing authors. Most of them originate from a neuroscientific orientation with only some contributions from the comparative and ethological approach. The common feature between all contributions was rigorous application of methods and inferences made with empirical data. As a whole, the topic seems to be timely, being exemplary of the increased interest toward music and emotion. There are, however, discrepancies in theories, observations, and approaches, as exemplified in the individual contributions.

This e-book is the outcome of this research topic. The bulk of contributions revolves around the specificity of music experience in terms of perception, emotional reactions, and aesthetic assessment. Since these constituents are also part of the experience of visual art, it seemed to be a fruitful strategy to analyze similarities and differences between these two modalities. As a whole, these studies present new data as well as new hypotheses and theoretical claims which can contribute to a better understanding of the functions of the brain as related to musical experience.

The contributions can be divided roughly in theoretical papers, empirical papers, and one applied paper. As to the theoretical papers, the contribution by Reybrouck and Eerola emphasizes the roots of the emotion induction and expression and provides a synthesis of a hierarchical framework of emotions spanning core affects, basic emotions and aesthetic emotions. Brattico et al. introduce a promising new framework to implement the statistical analysis of global sensory properties used in visual art into neuroaesthetical research of music. Other papers are also illustrative of a positive trend that there is more emphasis to attempt to come up with theories that would cover other domains than music alone. Tiihonen et al. concentrate on the conceptualization of pleasure elicited by music and visual-art in empirical studies. They provide a theoretical synthesis and demonstrate that pleasure is often an ill-defined term which is used differently in research on music and visual arts. Lee et al. compare the aesthetic experiences induced by music and visual stimuli by focusing on the cross-modal perception in the aesthetic experience of emotional visual music. They emphasize the differences in conveying emotional meaning between auditory and visual channel. The empirical papers, on the other hand, make up the bulk of the contributions. Gorzelańczyk et al. suggest that subcortical structures are involved in processing the syntax of music. Vuoskoski and Eerola promote the view that there are important individual differences in how people experience paradoxical emotions such as pleasure experienced during listening to sad music. Barrett and Schulkin argue on similar lines but stress also the role of granularity in processing emotional contents. Liang et al. highlight how the factors related to musical expertise provide easily measurable differences in pitch discrimination. Kuribayashi and nittono suggest that there are even more subtle reactions to audio, even to frequencies which are not assumed to carry much relevant information. And Arjmand et al. provide evidence that central markers of emotion, such as frontal asymmetry, are sensitive to high-level musical cues associated with positive affect. The contribution by Leubner and Hinterberger, finally, is the only example of an applied paper, and reviews the extant studies whether music intervention could significantly influence the emotional state of people with depression.

To our pleasure, several of the articles in this e-book have already been accessed thousands of times, indicating a genuine value of the novel angles, ideas, and findings offered within the contributions.

## Author contributions

All authors listed have made a substantial, direct and intellectual contribution to the work, and approved it for publication.

### Conflict of interest statement

The authors declare that the research was conducted in the absence of any commercial or financial relationships that could be construed as a potential conflict of interest.

